# Resilience of quantum spin fluctuations against Dzyaloshinskii–Moriya interaction

**DOI:** 10.1038/s41598-024-60502-y

**Published:** 2024-05-01

**Authors:** Saeed Mahdavifar, Mahboubeh Salehpour, Hadi Cheraghi, Kourosh Afrousheh

**Affiliations:** 1 Department of Physics, University of Guilan, Rasht, 41335-1914 Iran; 2 Computational Physics Laboratory, Physics Unit, Faculty of Engineering and Natural Sciences, Tampere University, FI-33014 Tampere, Finland; 3 Department of Physics, Kuwait University, P. O. Box 5969, 13060 Safat, Kuwait; 4 Helsinki Institute of Physics, University of Helsinki, FI-00014 Helsinki, Finland

**Keywords:** Quantum physics, Statistical physics, thermodynamics and nonlinear dynamics

## Abstract

In low-dimensional systems, the lack of structural inversion symmetry combined with the spin-orbit coupling gives rise to an anisotropic antisymmetric superexchange known as the Dzyaloshinskii–Moriya interaction (DMI). Various features have been reported due to the presence of DMIs in quantum systems. We here study the one-dimensional spin-1/2 transverse field XY chains with a DMI at zero temperature. Our focus is on the quantum fluctuations of the spins measured by the spin squeezing and the entanglement entropy. We find that these fluctuations are resistant to the effect of the DMI in the system. This resistance will fail as soon as the system is placed in the chiral phase where its state behaves as a squeezed state, suggesting the merit of the chiral phase to be used for quantum metrology. Remarkably, we prove that the central charge vanishes on the critical lines between gapless chiral and ferromagnetic/paramagnetic phases where there is no critical scaling versus the system size for the spin squeezing parameter. Our phenomenal results provide a further understanding of the effects of the DMIs in the many-body quantum systems which may be testable in experiments.

## Introduction

The ground state of a quantum many-body system determines its main characteristics. Hence, the efforts are focused on understanding how various interactions can change the ground state and create different phases separated by quantum critical points. These interactions can be of different types, such as short-range or long-range^1,2^, disorder or noise^3,4^, or non-Hermitian or complex^5,6^. In magnetic systems, the interplay of broken inversion symmetry and spin-orbit interaction, also called Dzyaloshinskii–Moriya interaction (DMI), can give favoring noncollinear chiral magnetic orders such as spin spirals and skyrmions^7–10^. The DMI is a type of interaction that can induce chirality in magnetic systems. It was first discovered in oxides^11^, and later found in other materials, such as Ir/Co/Pt- and Pt/Co/Ta-based heterostructures^12,13^, and multiferroic materials^14^. The DMI has many potential applications in spintronics, where the spin of electrons is used to transmit information^15^. However, the DMI also poses some challenges, such as understanding its origin and effect on the magnetic properties.

Quantum phase transitions are caused by the competition between different ground-state phases of a many-body system. Quantum information theory concepts, such as entanglement, have been widely used to identify quantum critical points in various complex many-body systems, as they capture the qualitative change in the collective many-body properties^16,17^. The relation between spin squeezing (SS) and entanglement has been a topic of much interest in the last two decades^18–21^. SS is a way of quantifying the quantum fluctuations of a spin system. It can also be used to detect and measure entanglement, which is a quantum correlation between different parts of a system. SS is a sufficient condition for entanglement in any system^18^, and there are general criteria based on SS to identify entangled states^19^. Moreover, SS implies that every pair of spins in a symmetric multiqubit state is entangled^20^. However, finding the exact amount of entanglement in complex quantum states is difficult, so SS can be a useful tool to estimate it^21^.

The total angular momentum components $J_{\alpha }$ of a system of *L* spin particles satisfy the commutation relation $[J_{\alpha },J_{\beta }]=i \hbar J_{\gamma }$, where $\alpha , \beta , \gamma$ are cyclic permutations of *x*, *y*, *z* and $J_{\alpha }=\sum _{n=1}^{L} S_{n}^{\alpha }$. This implies the uncertainty relation

1 $$\begin{aligned} (\Delta J_{\alpha })^{2} (\Delta J_{\beta })^{2} \ge |\langle J_{\gamma } \rangle |^{2}/4~, \end{aligned}$$

where $(\Delta J_\alpha )^2= \langle J_{\alpha }^2\rangle - \langle J_{\alpha } \rangle ^2$ is the variance and $\Delta J_{\alpha }$ is the standard deviation. In the standard quantum limit, a coherent state is a state of the system where $\Delta J_{\alpha }=\Delta J_{\beta }=\sqrt{|\langle J_{\gamma }\rangle |/2}$^22^. As soon as $\Delta J_{\alpha }$ or $\Delta J_{\beta }$ reduces below the standard quantum limit, a spin-squeezed state emerges which means that the quantum spin fluctuations of one of the total angular momentum components are smaller than those of the others^23–26^. For this reason, SS is applied in quantum metrology^27,28^ and optical atomic clocks^29,30^ to make it possible to do high-precision measurements.

The ground states of quantum systems with a mass gap and short-range interactions have a special property: their entanglement entropy grows logarithmically with the size of the subsystem. The coefficient of this growth is proportional to the central charge, a parameter that characterizes the conformal field theory that describes the system near a quantum critical point^31,32^. The entanglement entropy measures how much the quantum states of different parts of the system are correlated. This leads to new universality behaviors of quantum systems at critical points. On the contrary, the noncritical points unveil an exponentially decaying of the EE, which satisfies an “area law”. These features indeed can be violated for long-range interactions^33,34^, fractal entanglement phases^35^ as well as non-Hermitian models^36^, and for a given short-range interaction, i.e., DMI, as we will show.

We here consider the one-dimensional (1D) spin-1/2 XY chains in the presence of the transverse field (TF) and DMI, which is a mechanism for weak magnetism in some antiferromagnetic crystals^37,38^, with an application in quantum work engines^39^. The Hamiltonian of the model can be diagonalized by the Jordan-Wigner transformation^40–42^. The anisotropic model has three different phases in its ground state: a gapless phase with chirality, a gapped phase with ferromagnetism, and a gapped paramagnetic phase. These phases can be detected by using measures of quantum correlations, such as concurrence and quantum discord^42^. They can also be revealed by studying the work and entropy production when the system is suddenly quenched out of equilibrium^43^. Moreover, it is shown that the Berry phase of the isotropic system changes significantly with the DMI and the TF^44^. In Ref.^45^ although the authors studied the effect of the DMI on the quantum speed limit and orthogonality catastrophe under sudden quantum quenches, their results spontaneously affirmed the robustness of the initial quantum spin fluctuations against the DMI in a nonequilibrium quantum system which can be also interpreted as the persistence of the initial state versus dynamical phases^46,47^. This resiliency also can be observed in the behavior of the spin-spin correlation functions in the equilibrium^48^.

In this paper, we investigate the effect of the DMI on quantum spin fluctuations measured by the SS parameter and EE in the exactly solvable XY model which is equivalent to the Kitaev chain^49^, a one-dimensional topological superconductor. We obtain the ground-state phase diagram for the SS and the EE and study the behavior of these two parameters on the quantum critical lines as well as within phases. We show that the critical SS is not always extreme showing that on the quantum critical lines, the quantum fluctuations may not be exactly suppressed. We also find that the SS parameter on the critical line separating the gapped FM and PM phases scales asymptotically as the Heisenberg limit in infinite chains and as the standard quantum limit in finite chains. Moreover, our results for central charge expose that it will have zero value on the critical lines between the chiral phase with the FM and PM phases while within the chiral phase, it is one, introducing the chiral phase as a critical area.

The paper is organized as follows. In Sect. "The model", we introduce the model and using the fermionization approach the ground state of the system will be obtained. In Sect. "Spin squeezing and entanglement entropy", the SS parameter and EE will be introduced. In Sect. "Results", we represent our results on the behavior of the SS and EE in the whole range of the ground state phase diagram. We conclude and summarize our results in Sect. "Conclusions".

**Figure 1:**
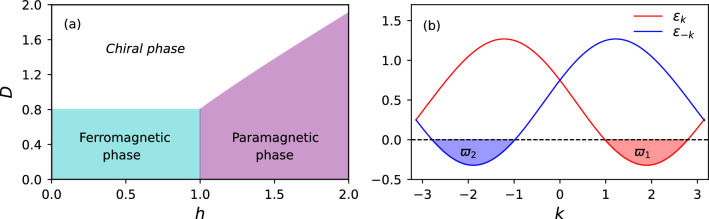
(**a**) The ground state phase diagram of the model for $\delta =0.8$. Three phases with three critical lines are observed. (**b**) The quasiparticle energy spectra $\varepsilon _{\pm k}$ in the chiral phase. The energy spectra will be negative in the regions $\varpi _1=[k_F^-,k_F^+]$ for $\varepsilon _k$ and $\varpi _2=[-k_F^+,-k_F^-]$ for $\varepsilon _{-k}$. Thus, one can write $\varpi _1=-\varpi _2=\varpi$.

## The model

We consider the spin-1/2 TF XY chain model with a DMI whose Hamiltonian is given by

2 $$\begin{aligned} {{\mathscr {H}}} = -J\sum \limits _{n = 1}^L {\left[ {(1 + \delta )S _n^x S _{n + 1}^x + (1 - \delta ) S _n^y S _{n + 1}^y} \right] } +D \sum \limits _{n = 1}^L (S _n^x S _{n + 1}^y-S _n^y S _{n + 1}^x)-h\sum \limits _{n = 1}^L {S _n^z}~, \end{aligned}$$

where $S_n$ is the spin operator on the *n*-th site, $J>0$ is the FM exchange coupling, *D* is the strength of the DMI, $0< \delta < 1$ is the anisotropy parameter, *h* is the homogeneous TF, and *L* is the system size (or a number of spins). We use the periodic boundary condition $S_{L+1}^\mu =S_1^\mu$ ($\mu =x,y,z$) and set $J=1$ without loss of generality. The ground state phase diagram of the model (see Fig. 1a) has three phases that are separated by critical lines as follows:

1. When $D<D_c=\delta$, the critical line is $h_c=1.0$, which separates the gapped FM and PM phases.
2. When $h<h_c=1$, the critical line is $D_c=\delta$, which separates the gapped FM phase from the gapless chiral phase.
3. When $D>D_c=\delta$, the critical line is $h_c=\sqrt{1+D^2-\delta ^2}$, which separates the gapped PM from the gapless chiral phases.

We diagonalize the Hamiltonian by using the Jordan-Wigner transformation^50^,

3 $$\begin{aligned} S^{+}_{n}=a_{n}^{\dag }e^{i\pi \sum ^{n-1}_{m=1}a^{\dag }_{m}a_{m}},~~~ S^{-}_{n}=e^{-i\pi \sum ^{n-1}_{m=1}a^{\dag }_{m}a_{m}}a_{n},~~~ S^{z}_{n}=a_{n}^{\dag }a_{n}-\frac{1}{2}, \end{aligned}$$

which maps the spin operators to fermionic operators $a_n^\dagger$ and $a_n$, and gives

4 $$\begin{aligned} {{\mathscr {H}}} = \sum \limits _{n = 1}^L \left[ \frac{(-1+iD)}{2} a_n^\dag a_{n + 1}- \frac{\delta }{2} a_n^\dag a_{n + 1}^\dag + h.c. \right] - h\sum \limits _{n = 1}^L a_n^\dag {a_n}. \end{aligned}$$

then, applying Fourier transformation ${a_n} = (1/\sqrt{L})\sum _k e^{ ikn} {a_k}$ and Bogoliuobov transformation ${a_k} = \cos ({\theta _k}) {\beta _k} + i \sin ({\theta _k}) \beta _{- k}^{\dag }$ lead to obtain the diagonalized Hamiltonian

5 $$\begin{aligned} {{\mathscr {H}}} = \sum \limits _k {\varepsilon }_k \left[ {\beta _k^\dag {\beta _k} - \frac{1}{2}} \right] , \end{aligned}$$

with energy spectrum $\varepsilon _k ={{\mathscr {B}}}_k+ \sqrt{{{\mathscr {A}}}_k ^2+{{\mathscr {C}}}_k ^2}$ where ${{\mathscr {A}}}_k= -[\cos (k)+h]$, ${{\mathscr {B}}}_k= -D \sin (k)$ and ${{\mathscr {C}}}_k= - \delta \sin (k)$ are related to the Bogoliubov angle $\theta _k$ by $\tan (2{\theta _k}) =- {\mathscr {C}}_k/{{\mathscr {A}}}_k$. The summation in Eq. (5) runs over $k=2\pi m/L$, with $m=0,\pm 1,...,\pm \frac{1}{2}(L-1) \ [m= 0, \pm 1,..., \pm (\frac{1}{2}L-1), \frac{1}{2}L]$ for odd [even] *L* (using periodic boundary conditions for the Jordan-Wigner fermions). As we see, although DMI changes the energy spectrum of the system, but has no effect on the Bogoliubov angle. In this setting, a phase transition due to gap-closing originating from DMI will not happen in the system. Consequently, its effect just appears when the system is put in the chiral phase, which creates a phase transition and leads to variations in the Fermi points. One can in general read the Fermi points satisfying the condition $\varepsilon _k < 0$ by the way of

6 $$\begin{aligned} k_F^ \pm = \arccos \left[ \frac{ h \mp \sqrt{({D^2} - {\delta ^2})(1 +D^2-\delta ^2-h^2)}}{1+D^2-\delta ^2} \right] \end{aligned}$$

In addition, one can rewrite Eq. (5) as ${{\mathscr {H}}} = (1/2) \sum _k [\varepsilon _k \beta _k^\dag \beta _k+\varepsilon _{-k} \beta _{-k}^\dag \beta _{-k} - (\varepsilon _k+\varepsilon _{-k})/2 ]$. In Fig. 1b we indicated $\varepsilon _{\pm k}$ for a case where it is located in the chiral phase. As seen, in this situation in two regions we face $\varepsilon _{\pm k}<0$, as $\varpi _1=[k_F^-,k_F^+]$ for $\varepsilon _k$ and $\varpi _2=[-k_F^+,-k_F^-]$ for $\varepsilon _{-k}$ where $\varpi _1=-\varpi _2=\varpi$. This helps us to write the ground state of the system in a general form of^51^

7 $$\begin{aligned} | GS\rangle = \left( {\prod \limits _{k \notin {\varpi _1},{\varpi _2}} {| {0_k},{0_{ - k}}\rangle } } \right) \otimes \left( {\prod \limits _{{\varpi _1}} {\beta _k^\dag | {0_k},{0_{ - k}}\rangle } } \right) \otimes \left( {\prod \limits _{{\varpi _2}} {\beta _{ - k}^\dag | {0_k},{0_{ - k}}\rangle } } \right) \end{aligned}$$

since we need to calculate the required parameters in the ground state of the system. Here $| {0_k},{0_{ - k}}\rangle$ is vacuum of the Bogoliubov as $\beta _{\pm k}| {0_k},{0_{ - k}}\rangle =0$.

## Spin squeezing and entanglement entropy

We use the SS parameter defined by^23^

8 $$\begin{aligned} \xi _s^2 = \frac{4(\Delta J_{\textbf{n}_{\perp }})^2}{L}, \end{aligned}$$

where $\textbf{n}_{\perp }$ is an axis perpendicular to the average spin direction $\textbf{n}_0 = \langle \textbf{J} \rangle /|\langle \textbf{J} \rangle |$, and the variance $(\Delta J)^2$ is minimized, with $J_{\textbf{n}_{\perp }} = \textbf{J}\cdot \textbf{n}_{\perp }$. We note that there is another SS parameter given by $\xi _R^2 = L(\Delta J_{\textbf{n}_{\perp }})^2/ | \langle J_{\textbf{n}} \rangle |$ that was introduced by Wineland^24,25^.

The coherent state corresponds to $\xi _s^2 =1$, with the inequality $\xi _s^2 <1$ indicating that the system is in a SS state. Considering the symmetries of the model as the unbroken $Z_2$ invariance for finite *L* implies that $\langle J_x \rangle = \langle J_y \rangle = 0$, and similarly,

9 $$\begin{aligned} \langle {J_\alpha J_z} \rangle = \langle {J_z J_\alpha }\rangle = 0, ~~~~ \alpha = x,y. \end{aligned}$$

The magnetization for $h>0$ is always along the *z*-axis, with full polarization developing in the PM phase. As a result, $J_{\textbf{n}_{\perp }} = \cos (\Omega )J_x + \sin (\Omega )J_y$, with $\Omega$ to be chosen to minimize

10 $$\begin{aligned} (\Delta {J_{\textbf{n}_{\perp }}})^2 = \langle (J_ {\overrightarrow{n} \bot } )^2 \rangle - \langle J_ {\overrightarrow{n} \bot } \rangle ^{2} \end{aligned}$$

One can easily show that^52,53^

11 $$\begin{aligned} \xi _s^2= & {} \frac{2}{L}\mathop {\min }\limits _\Omega \big ( \langle J_x^2 + J_y^2\rangle + \cos (2\Omega )\langle J_x^2 - J_y^2\rangle \big ) \nonumber \\= & {} \frac{2}{L} \big [\langle J_x^2 + J_y^2\rangle -\sqrt{\langle J_x^2 - J_y^2 \rangle ^{2}+ \langle {J_x J_y + J_y J_x} \rangle ^{2}} \big ]. \end{aligned}$$

A key quantity entering the characterization of entanglement is the entanglement entropy (EE) described by bipartite^54^ and disconnected^55^ partitions, which is widely used in quantum information theory. The former case is defined as the von Neumann entropy of a reduced density matrix of a subsystem^56–58^ so that for the pure ground state $|\psi \rangle$ with the density matrix $\rho =|\psi \rangle \langle \psi |$, it is expressed by

12 $$\begin{aligned} S_{A}=-{{\textrm{Tr}}}[ \rho _{A} \log _{2} (\rho _{A})] \end{aligned}$$

where $\rho _{A}= {{\textrm{Tr}}}_{B} (\rho )$ is the reduced density matrix of *A* obtained by tracing over the rest of the system *B*. The EE usually grows like the boundary area of the subsystem *A*, and not like its volume, which is different from expected extensive behavior, known as the “area law”, with an extensive study in recent years. Noncritical ground-states of spin chains with a finite correlation length have a constant EE. At a quantum critical point, when subsystem *A* is a finite interval of length *L*/2, the EE slightly violates the area law by a logarithmic correction as, $S_{L/2} (L) = (c_{eff}/3) \log (L)+b$, where $c_{eff}$ is the central charge^31,32^, and *b* is a non-universal constant. In general, the EE of a finite block of $l_b$ sites in an infinite system of free spinless fermions can be computed by^59^

13 $$\begin{aligned} S_{A}=-\sum \limits _{j = 1}^{2l_b} \lambda _j \log (\lambda _j), \end{aligned}$$

where $\lambda _j$ are the eigenvalues of the $2l_b \times 2l_b$ correlation matrix ${\mathscr {M}}$ constructing as

14 $$\begin{aligned} {{\mathscr {M}}} = \left( {\begin{array}{*{20}{c}}P&{}Q\\ {{Q^\dag }}&{}{R}\end{array}} \right) \end{aligned}$$

where *F*, *Q* and *R* are $l_b \times l_b$ matrices build up by two-point correlators $P_{nm} = \langle a_n^\dag {a_m}\rangle$, $Q_{nm} =\langle {a_n^\dag a_m^\dag }\rangle$, and $R_{nm} =\delta _{n,m}-P_{mn}$ respectively. The two-point correlators are calculated through

15 $$\begin{aligned} P_{nm}= & {} \frac{\delta _{nm}}{2} - \frac{1}{2L}\sum \limits _{k} {\cos [k(m - n)]\cos (2{\theta _k})} + \frac{1}{L}\sum \limits _{k \in \varpi } \left\{ \cos [k(m - n)]\cos (2\theta _k)+i\sin [k(m-n)]\right\} \nonumber \\ Q_{nm}= & {} \frac{1}{2L}\sum \limits _{k} {\sin [k(m - n)]\sin (2\theta _k)} - \frac{1}{L}\sum \limits _{k \in \varpi } \sin [k(m - n)]\sin (2\theta _k) \end{aligned}$$

in which $\varpi$ denotes a *k*-space region with $\varepsilon _k<0$, and *L* is the total system size. With these two in hand, we can now calculate the required parameters to obtain the SS parameter. By introducing $A_r = a_r^\dagger + a_r$ and $B_r = a_r^\dagger - a_r$, and also $G_{r}^{\alpha \beta }$ that denotes two-point correlation functions, a direct calculation shows that

16 $$\begin{aligned} G_r^{xx } = \langle S_1^xS_{1 + r}^x \rangle = \frac{1}{4}\langle B_1 A_2B_2...A_rB_rA_{r+1} \rangle ~~&;&~~ G_r^{yy} =\langle S_1^yS_{1+r}^y \rangle = \frac{( - 1)^r}{4}\langle A_1B_2A_2...B_rA_rB_{r+1} \rangle , \nonumber \\ G_r^{xy } =\langle S_1^xS_{1 +r}^y \rangle = \frac{ - i}{4}\langle B_1A_2B_2...A_rB_rB_{r+1} \rangle ~~&;&~~ G_r^{yx } =\langle S_1^yS_{1 + r}^x \rangle = \frac{ i( - 1)^r}{4}\langle A_1B_2A_2...B_rA_rA_{r+1} \rangle , \end{aligned}$$

These equations may be written in the generic form, $G_r^{\alpha \beta } = D_r^{\alpha \beta } \langle {{\phi _1}{\phi _2}{\phi _3}...\phi _{2r-2}\phi _{2r-1}{\phi _{2r}}} \rangle$, with

17 $$\begin{aligned} D_r^{xx} = \frac{1}{4}~~;~~ D_r^{yy} = \frac{( - 1)^r}{4}~~;~~ D_r^{xy} = \frac{ - i}{4}~~;~~ D_r^{yx} = \frac{i( - 1)^r}{4}, \end{aligned}$$

where each operator $\phi _j, j= 1,2, \ldots , 2r$, is identified with either an $A_r$ or a $B_r$ operator. Using the Wick theorem^60^, the 2*r*-point functions can be expressed as Pfaffians

18 $$\begin{aligned} G_r^{\alpha \beta } = D_r^{\alpha \beta }pf\left( {\begin{array}{*{20}{c}} {\langle {{\phi _1}{\phi _2}} \rangle }&{}{\langle {{\phi _1}{\phi _3}}\rangle }&{}{\langle {{\phi _1}{\phi _4}} \rangle }&{} \cdots &{}{\langle {{\phi _1}{\phi _{2r}}} \rangle }\\ {}&{}{\langle {{\phi _2}{\phi _3}} \rangle }&{}{\langle {{\phi _2}{\phi _4}}\rangle }&{} \cdots &{}{\langle {{\phi _2}{\phi _{2r}}}\rangle }\\ {}&{}{}&{}{\langle {{\phi _3}{\phi _4}}\rangle }&{} \cdots &{}{\langle {{\phi _3}{\phi _{2r}}} \rangle }\\ {}&{}{}&{}{}&{} \ddots &{} \vdots \\ {}&{}{}&{}{}&{}{}&{}{\langle {{\phi _{2r - 1}}{\phi _{2r}}} \rangle } \end{array}} \right) \end{aligned}$$

where we have written the skew-symmetric matrix in the standard abbreviated form. Our calculations unveil that

19 $$\begin{aligned} \langle A_nA_m \rangle= & {} -\langle B_nB_m \rangle =\delta _{nm}+\frac{2i}{L}\sum \limits _{k \in \varpi } \sin [k(m-n)] \nonumber \\ \langle A_nB_m \rangle= & {} \frac{1}{L}\sum \limits _{k} {\cos [k(m - n) - 2{\theta _k}]} - \frac{2}{L}\sum \limits _{k \in \varpi } {\cos [k(m - n) - 2{\theta _k}]} \nonumber \\ \langle B_nA_m\rangle= & {} - \frac{1}{L}\sum \limits _{k} {\cos [k(m - n) + 2{\theta _k}]} + \frac{2}{L}\sum \limits _{k \in \varpi } {\cos [k(m - n) + 2{\theta _k}]} \end{aligned}$$

In addition, on the one hand,

20 $$\begin{aligned} \langle J_xJ_y\rangle = \sum \limits _{r = 1}^L {\langle {S_r^xS_r^y} \rangle } + \sum \limits _{r = 1}^{L - 1} {(L- r)G_r^{xy}} + \sum \limits _{r = 1}^{L - 1} {r~G_{L-r}^{yx}}~~~~;~~~~ \langle J_yJ_x\rangle = \sum \limits _{r = 1}^L {\langle {S_r^yS_r^x} \rangle } + \sum \limits _{r = 1}^{L - 1} {(L - r)G_r^{yx}} + \sum \limits _{r = 1}^{L - 1} {r~G_{L-r}^{xy}} \end{aligned}$$

and on the other hand, from the Hamiltonian (2) one can extract that $\langle S_n^xS_m^y\rangle =-\langle S_n^yS_m^x\rangle$. Hence, $\langle J_xJ_y+J_yJ_x\rangle =0$. Accordingly, the SS parameter is obtained by

21 $$\begin{aligned} \xi _s^2 = 1+2 \sum \limits _{r = 1}^{L-1}(G_r^{xx}+G_r^{yy})-2|\sum \limits _{r = 1}^{L-1}(G_r^{xx}-G_r^{yy})|, \end{aligned}$$

**Figure 2:**
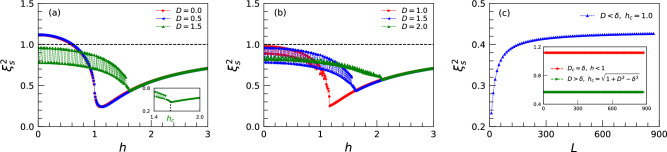
(**a**) and (**b**) belong to the SS parameter versus TF for different values of DMI and a chain with size $L = 200$. In contrast to the FM and PM phases, as explicitly seen, the effective size in the chiral phase is intense. The horizontal black dashed line is for $\xi _s^2=1$. The inset in (**a**) clearly shows the critical TF where the system is put in the PM phase that for $D=1.5$ it happens at $h_c \approx 1.62$. (**c**) The SS parameter as a function of the chain size *L* on the critical line $h_c = 1$ for $D<\delta$ where the gapped FM and PM phases are separated. The inset in (**c**) shows the lack of scaling for the other two quantum critical lines. Here and also in other figures, we fix $J=1.0$, $\delta =0.8$.

## Results and discussions

We first study how the SS parameter changes with the TF and DMI in the lowest energy state of the system, for the case $\delta =0.8$. The results for other values of the anisotropy as $0< \delta <1$ are similar.

As seen in Fig. 2a, in the absence of the TF, the ground state of the system can be an unsqueezed state in the FM phase ($D\le D_c$) or a squeezed state in the chiral ($D\ge D_c$) phase. This reveals that the quantum systems with chiral phases in their ground states are good candidates for quantum metrology^61^. By applying TF, the SS parameter shows a decreasing behavior up to the extreme SS value for $D<D_c$ occurring slightly after critical TF $h_c=1$, or a quantum critical TF $h_c$ for $D\ge D_c$. This means that the extreme point is not only a property of the PM phase. In contrast, it depends on phases on both sides of a given critical line. However, with more increasing TF, the SS parameter behaves inversely. In the large value of the TF, the SS parameter tends to have a value near one, to be a coherent state.

The crossover between squeezing and non-squeezing emerges in the FM phase at the value of $\xi _s^2=1$ where supports a coherent state at the factorized point $h=\sqrt{1-\delta ^2}$^62^. This is because, at this point, the ground state becomes twofold-degenerate again due to a finite size effect^63^. Therefore, in general, in the ground state phase diagram, the PM and the chiral phases are always squeezed. The ground state is unsqueezed only in the region $D<\delta$ and $h<\sqrt{1-\delta ^2}$ of the FM phase. As we obviously viewed in Fig. 2a and b, the FM and PM phases unveil robustness versus the impact of DMI, independent of its amount, as all values of the SS parameter are the same in these phases. Resistance is broken and a response will be exposed as soon as the system is located in the chiral phase where the quantum spin fluctuations are dependent on the DMI. See Fig. 2b. Note that all fluctuations in the chiral region are merely finite-size effects. To get a confirmation, in Supplementary Fig. 1a, we plotted the SS parameter for size $L=800$ where we explicitly indicated a reduction of the fluctuations by increasing the system size.

**Figure 3:**
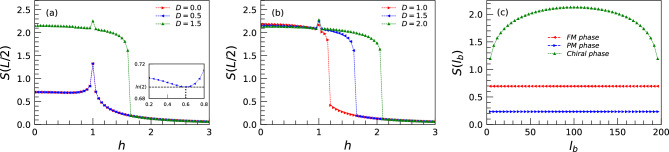
(**a**) and (**b**) correspond to the EE versus TF for different values of DMI. As obvious, the effective size for EE in the chiral phase is around $h=1$ as a jump. The inset in (**a**) clearly illustrates the value of the EE in the coherent state, $h=0.6$, as $S(L/2)= \ln (2)$. (**c**) is for the EE as a function of the different subsystems $l_b$ within the three phases. Here sizes of all chains are fixed to $L = 200$.

Finally, we calculated the quantum critical SS at $h_c=1$ and $D<D_c$ i.e., on the critical line between the FM and PM phases, for different system sizes. The results are shown in Fig. 2c, clearly illustrating that the quantum critical SS increases with the system size *L*. We found two different scaling behaviors depending on the system size. For very large systems the critical SS parameter scales in the form of

22 $$\begin{aligned} \xi _s^2\sim L^{-1/2}, \end{aligned}$$

which is known as the standard quantum limit. Based on the Heisenberg limit, precision scales no better than $L^{-1/2}$ with the total number of probes used in an experiment^64–66^. For small systems, the critical SS parameter scales linearly with inverse system size as,

23 $$\begin{aligned} \xi _s^2\sim L^{-1}, \end{aligned}$$

in compliance with the Heisenberg limit^67,68^. The standard quantum limit is the bound on the sensitivity that can be achieved by using classical states such as coherent states. Notably, as is illustrated in the inset in Fig. 2c, for the other two critical lines, no scaling is observed, $\xi _s^2 \propto {{\mathscr {O}}}({L^0})$. Furthermore, it is noteworthy to mention that the value of the SS parameter within the FM and PM phases are independent of the system size, contrary to the chiral phase where its value is affected by the size of the system although goes to a constant value at the thermodynamics limit. See in Supplementary Fig. 1c.

Ramsey interferometers can use entangled particles to achieve higher metrological sensitivity, as shown by some quantitative relations^69,70^. By manipulating the interactions among the particles, one can create entangled multipartite quantum states that are suitable for enhancing interferometric measurements. To distinguish between different kinds of entangled multipartite states, one can use the EE as a measure^71^. The EE indicates how much information is missing when one part of the system is ignored and only the rest is observed^72^. Moreover, the EE can reveal how the entanglement between different regions of a quantum system varies across different phases with signaling on phase boundaries.

In this part, we study the EE in two scenarios: (i) fixing the system size to a given value *L* and computing the EE for different subsystems $l_b$, (ii) calculating the EE for different system sizes where the subsystem for each size is the half of the given chain, $l_b=L/2$. For the former case, it is illustrated that for the anisotropic XY model at the critical lines^73^, $S(l_b)$ will be maximized at the $l_b=L/2$. In addition, the central charge at the critical line between the FM and PM phases is 0.5, and the rest is zero. Moreover, in systems with a gap, the $S_{L/2}(L)$ quickly reaches a constant value, which is called the “area law”. In continuing, we investigate three critical lines as well as points within three phases.

**Figure 4:**
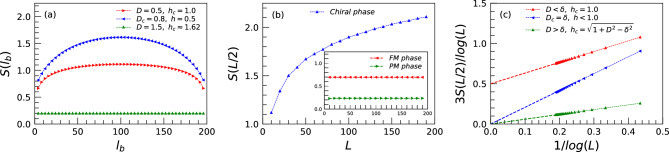
(**a**) is for the EE as a function of the different subsystems $l_b$ on the three critical lines for a system with size $L=200$. (**b**) is for the EE as a function of the chain size *L* within the three phases. (**c**) is for the EE divided by the logarithm of the system size *L* versus $1/\log (L)$ on three critical lines. The effective central charge $c_{eff}$ can be read out from the intercept, $3S_{L/2}(L)/\log (L)=b/\log (L)+c_{eff}$.

In Fig. 3a and b, we have presented our results on $S_{L/2}(L)$ for a chain size system $L=200$ for different values of DMI. As reflected, the chiral phase of the system shows more entanglement than the FM and PM phases. Moreover, signals on the critical points unveil the ability of the EE to detect quantum phase transitions. Although there is a sudden jump at $h=1$ in the chiral phase, it originates from the effective size and thus will vanish in high system sizes (see in Supplementary Fig. 1b). The inset in Fig. 3a shows the results of EE in the region close to a coherent state. The coherent state in this model is a separable state, so the EE should be zero. However, it is $\ln (2)$ instead of zero. This is because the ground state of the system at $h=0.6$ does not break any symmetry in a finite-size system, and it has the form $|GS\rangle = (| \uparrow \downarrow \uparrow \downarrow \cdots \rangle + | \downarrow \uparrow \downarrow \uparrow \cdots \rangle )/\sqrt{2}$, which gives the value $\ln (2)$ for the EE. We again can conclude that the FM and PM phases are robust against the presence of DMI. On the other hand, Fig. 3c exposes an exotic behavior of the EE within the chiral phase when different subsystems are considered. In the cases of the FM and PM phases, the EE for each one has a constant value, independent of the subsystem sizes while it changes for the chiral phase, and becomes maximized at $l_b=L/2$. This behavior is the same as the critical lines in the anisotropic XY model. For this reason, one can claim that the chiral phase is a critical region^48^.

The evolution of the EE with respect to the different subsystem sizes for a fixed size $L=200$ when the system is on the critical lines is shown in Fig. 4a. As expected for the anisotropic XY model, we see changes in the EE versus $l_b$ on the critical lines between the FM phase with the PM phase and also the chiral phase, all are maximized at $l_b=L/2$. In contrast, the EE on the critical line between the PM and chiral phases does not feel any effects when $l_b$ changes. In order to detect the values of the central charge within the phases as well as the critical lines, in Fig. 4b and c, we focus on the behavior of the $S_{L/2}(L)$ for different system sizes. As obvious, the EE only changes within the chiral phase, revealing $c_{eff}=1.0$ while its values keep constant within the FM and PM phases, indicating for both $c_{eff}=0.0$. On the other side, on the critical lines, the central charge tends to be 0.5 for $h_c=1$, $D<\delta$, the same as the anisotropic XY model, while becomes zero on the other critical lines which separate the chiral phase with the FM or the PM phases.

**Figure 5:**
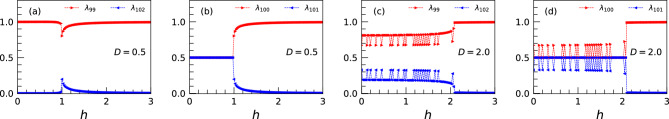
Entanglement spectrum versus the TF for a system with size $L=200$ as (**a**) and (**c**) $\lambda _{99}$, $\lambda _{102}$, and (**b**) and (**d**) for $\lambda _{100}$, $\lambda _{101}$. Furthermore, (**a**) and (**b**) are for $D=0.5$, and (**c**) and (**d**) are for $D=2.0$.

It should be noted that in systems with long-range interactions, the central charge can depend on the decay exponent of the interactions and can deviate from the short-range value. For example, in the long-range Kitaev chains, the central charge is 0.5 on the critical line and zero within the phases with short-range interactions, but it becomes 1.0 on the other critical lines and 0.5 within the phases with long-range interactions^74^. Here, we explicitly showed that the central charge can be also zero on critical lines or one within phases even in systems with short-range interactions.

The knowledge of the correlation matrix enables us to calculate the entanglement spectrum. The entanglement spectrum is a generalization of the entanglement entropy, which quantifies the quantum correlations between different parts of a system obtained by the set of eigenvalues of the reduced density matrix of a subsystem, which is a trace out the rest of the system. It can reveal important information about the quantum phases, transitions, and dynamics of the system, as well as its topological order and symmetry properties^75,76^. We here studied the entanglement spectrum for some eigenvalues of the correlation matrix shown in Fig. 5. The size of the chain is $L=200$ and we plotted the results for two values of the DMI as $D=0.5$, and $D=2.0$. For the former case, the TF makes a phase transition from the FM to PM phases. On the contrary, for the latter case, the TF constructs a phase transition from the chiral to PM phases. In both situations, as seen, the power of the entanglement spectrum on signaling at the critical points is clear. As evidence, two middle eigenvalues of the correlation matrix, $\lambda _{100},~\lambda _{101}$, are degenerate at the FM and chiral regions where the topological phases emerge resulting in degeneracies of low-lying entanglement spectrum. In this situation, the low-lying entanglement spectrum will be 1/2. Hence, the entanglement spectrum is able to detect the topological phase^77^.

## Conclusion

We investigated the zero-temperature behavior of the 1D spin-1/2 TF XY model with the DMI using the SS parameter and the EE. The model has a rich ground state phase diagram, with gapped FM and PM phases and a gapless chiral phase separated by three quantum critical lines for the anisotropic case. We computed the ground state SS and EE for the whole phase diagram and showed that they can detect all the quantum critical lines. We directly indicated that the FM and PM phases do not respond to the presence of the DMI. As soon as the system is located in the chiral phase, the effects of the DMI in the system emerge. Albeit in the systems with finite size, the quantum fluctuations will arise in the chiral phase but in the thermodynamics limit, one can participate they will fade.

We found that the quantum spin fluctuations are not critically suppressed on the quantum critical line that separates the gapped FM and PM phases. On this line, the critical SS parameter scales as the Heisenberg limit ($\sim L^{-1/2}$) for an infinite-size system and as the standard quantum limit ($\sim L^{-1}$) for finite-size chains.

We also studied the EE on the critical lines and within the phases. Our outcomes disclosed that it does not deviate from the area law on the critical line that separates the gapless chiral and FM or PM phases. On this line, the central charge is zero. Interestingly, within the chiral phase, a central change with $c_{eff}=1.0$ appears showing a critical behavior of this phase. On the other hand, the central charge is 0.5 on the critical line between the gapped FM and PM phases, which is the same as the anisotropic XY chain model. We also illustrated that the entanglement spectrum can identify quantum critical lines and topological phases in the system.

We hope that our work will stimulate further research on the critical SS parameter and EE in other 1D spin systems with short-range interactions and attract the opinion of researchers in the field of quantum metrology to the quantum systems including gapless phases.

### Supplementary Information

Supplementary Information.

## Data Availability

The data sets used and/or analyzed during the current study are available from the corresponding author upon reasonable request.
