# Supplementary material for: Resilience of quantum spin fluctuations against Dzyaloshinskii–Moriya interaction
Source: Sci Rep. 2024 May 1;14:10034. doi: 10.1038/s41598-024-60502-y (PMC11063192; doi:10.1038/s41598-024-60502-y)
Supplement: Supplementary file 1 — Supplementary Information. [file 41598_2024_60502_MOESM1_ESM.pdf]

# Supplementary Material

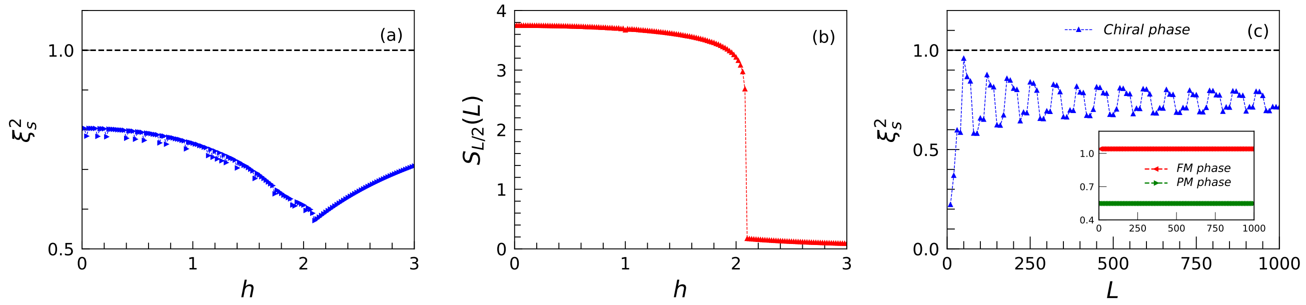


**Figure 1.** (color online). (a) The SS parameter and (b) the EE against the TF for $D = 2.0$ and a system with size $L = 800$. As viewed, in a high system size the fluctuations in the chiral phase reduce. Our results show that both parameters are valuable ways of exploring quantum critical lines even in systems with gapless phases. (c) is plotted for the SS parameter versus different system sizes for points within three phases as $D = 2.0,\ h = 0.5$ for the chiral phase, $D = 0.5,\ h = 0.5$ for the FM phase, and $D = 0.5,\ h = 2.0$ for the PM phase. The results unveil the ineffectiveness of the SS parameter rather than changing size in the FM and PM phases, $\xi_s^2 \propto \mathscr{O}(L^0)$. Remarkably, the changes in the chiral phase for small sizes are severe but in high sizes, the fluctuations decrease. This means one can accept at the thermodynamics limit, all of them disappear.
